# Replacement names and nomenclatural comments for problematic species-group names in Europe's Neogene freshwater Gastropoda. Part 2

**DOI:** 10.3897/zookeys.429.7420

**Published:** 2014-07-29

**Authors:** Thomas A. Neubauer, Mathias Harzhauser, Andreas Kroh, Georgopoulou Elisavet, Oleg Mandic

**Affiliations:** 1 Geological-Paleontological Department, Natural History Museum Vienna, 1010 Vienna, Austria

**Keywords:** Homonyms, synonyms, nomina nova, fossil freshwater gastropods

## Abstract

In the course of a new database project on Miocene to Recent freshwater gastropods of Europe, a great many of primary and secondary homonyms were revealed. Such nomenclatural issues need clarification in order to avoid misunderstandings and wrong statements about geographical distributions and temporal ranges. The following 16 new names are introduced to replace existing homonyms: *Theodoxus militaris jurisicpolsakae* **nom. n.**, *Viviparus stevanovici* **nom. n.**, *Melanopsis haueri ripanjensis* **nom. n.**, *Melanopsis wolfgangfischeri* **nom. n.**, *Micromelania ramacanensis* **nom. n.**, *Pseudamnicola welterschultesi* **nom. n.**, *Muellerpalia haszprunari* **nom. n.**, *Muellerpalia pseudovalvatoides* **nom. n.**, *Lithoglyphus gozhiki* **nom. n.**, *Valvata heidemariae willmanni* **nom. n.**, *Radix macaleti* **nom. n.**, *Gyraulus okrugljakensis* **nom. n.**, *Gyraulus rasseri* **nom. n.**, *Gyraulus vrapceanus* **nom. n.**, *Planorbarius halavatsi* **nom. n.**, and *Segmentina mosbachensis* **nom. n.** Additionally, six cases of homonyms are discussed that are not replaced by new names, because they are considered junior synonyms.

## Introduction

The latest estimate of living freshwater gastropod species involves about 4,000 described and valid species world-wide (Strong et al. 2008). Including the names for fossil gastropods, which were not considered in that study, certainly doubles if not multiplies the estimation on introduced and formally available species-group names. The practice to use common, descriptive terms (e.g., "*carinatus*", "*rugosus*" or "*elongatus*") as species-group names resulted in a great number of primary and secondary homonyms.

As to the fossil part, there are several publications dealing explicitly with this problem. In four subsequent works Pallary (1916, 1920, 1925, 1926) compiled the existing names of fossil and Recent melanopsid species described up to that time and introduced many new names for numerous homonyms. Likewise, Wenz came across a great number of such homonyms for terrestrial and freshwater gastropods when gathering literature for his Fossilium Catalogus (Wenz 1923–1930). In a series of eleven short nomenclatural works, he disposed of such errors by introducing replacement names (Wenz 1919a, 1919b, 1919c, 1922, 1923a, 1924, 1925, 1928b, 1928c, 1929b, 1930).

The newly established FreshGEN (Freshwater Gastropods of the European Neogene) database project, an initiative aimed at a pan-European reconstruction of the Neogene and Quaternary biodiversity of lacustrine gastropods, successively uncovered nomenclatural mistakes that have not yet been detected and/or revised. Following the first part of the resulting nomenclatural amendment (Neubauer et al. 2014), the current paper settles newly disclosed conflicts by introducing replacement names where required. This contribution is certainly just a small part in a greater picture, but is an essential basis for any future studies. In almost all cases this regards primary homonyms; only for two secondary homonyms replacement names are established, where the generic attribution is considered reliable. Only those primary homonyms are replaced that are today considered accepted taxa, ergo not disused junior synonyms. Such cases as well as two apparent homonyms are additionally discussed.

The systematics follows Bouchet et al. (2005), Jörger et al. (2010), Criscione and Ponder (2013), and the WoRMS database. Where available, information about type locality, age of the deposits, and type material is taken from the original publications. In cases where this information is lacking, insufficient or wrong, other sources were consulted and are specified accordingly.

## Homonyms

### Class Gastropoda Cuvier, 1795
Subclass Neritimorpha Golikov & Starobogatov, 1975
Order Cycloneritimorpha Frýda, 1998
Superfamily Neritoidea Rafinesque, 1815
Family Neritidae Rafinesque, 1815
Subfamily Neritininae Poey, 1852

#### Genus Theodoxus

Montfort, 1810

##### Type species.

*Theodoxus lutetianus* Montfort, 1810 [currently considered as a synonym of *Theodoxus fluviatilis* (Linnaeus, 1758)]. Recent, Europe. Type by original designation (Welter-Schultes 2012, p. 26).

#### Theodoxus militaris jurisicpolsakae nom. n.

Theodoxus (Theodoxus) militaris oblongus Jurišić-Polšak, 1979: 28, pl. 10, fig. 2 [non *Neritina leobersdorfensis oblonga* Handmann, 1887].

##### Etymology.

In honor of Zlata Jurišić-Polšak (Croatian Natural History Museum), who contributed to our knowledge of Neogene Neritidae.

##### Type locality.

Malino, Croatia.

##### Age.

Late Pliocene to Early Pleistocene ("*Paludina* Beds").

##### Syntypes.

Croatian Natural History Museum, coll. no. 9454.

##### Discussion.

Handmann (1887, p. 9) described and figured *Neritina leobersdorfensis* var. *oblonga* from the Late Miocene of the Vienna Basin and made it thus available as species-group name (published before 1961, see ICZN 1999, Articles 45 and 57.1). The subspecific status was maintained by Papp (1953, p. 99), who recombined the species with *Theodoxus*. Although Jurišić-Polšak (1979) mentioned that species and cited both works in her study about Miocene and Pliocene neritids from Croatia, she established the name *oblongus* for a different species-group taxon in *Theodoxus*. Thereby she referred to a determination by Spiridion Brusina, who had established the name "*Neritina militaris* var. *oblonga*" for material in the collection but never published it. Jurišić-Polšak accepted this "*in schedis*"-determination and formally described the subspecies, obviously unaware of the fact that this would create a secondary homonym. It can be separated from the nominal species by the more elevated spire and the fewer axial ribs.

### Subclass Caenogastropoda Cox, 1960
Order unassigned
Superfamily Viviparoidea Gray, 1847
Family Viviparidae Gray, 1847
Subfamily Viviparinae Gray, 1847

#### Genus Viviparus

Montfort, 1810

##### Type species.

*Viviparus fluviorum* Montfort, 1810 [currently considered as a synonym of *Viviparus viviparus* (Linnaeus, 1758)]. Recent, Northern Eurasia, Europe, Anatolia and Northern America. Type by original designation (Welter-Schultes 2012, p. 31).

#### Viviparus stevanovici nom. n.

Viviparus elongatus Stevanović, 1978: 325, pl. 5, figs 1–3 [non *Paludina elongata* d'Orbigny, 1837].
Viviparus elongatus Stevanović; Stevanović 1990: 501, pl. 14, figs 9–10 [non d'Orbigny 1837].

##### Etymology.

In honor of Petar M. Stevanović (Belgrade), who greatly contributed to our knowledge of the mollusc fauna and biostratigraphy of the Late Miocene of Serbia.

##### Type locality.

Kostolac opencast mine, Serbia.

##### Age.

Late Miocene to Early Pliocene (Late Pannonian, Late Portaferrian).

##### Holotype.

Natural History Museum, Belgrade, coll. no. 5683.

##### Discussion.

Since *Paludina* Férussac, 1812 is a junior objective synonym of *Viviparus* Montfort, 1810 (ICZN 1959, Op. 573), this species is a primary homonym of the Late Eocene *Viviparus elongatus* (d'Orbigny, 1837) from the Paris Basin and needs a nomen novum. The Eocene species has been synonymized with the co-occurring *Hydrobia pyramidalis* (Férussac, 1814) by Sandberger (1873, p. 266), a decision followed by Wenz (1926, p. 1968).

### Order unassigned
Superfamily Cerithioidea Fleming, 1822
Family Melanopsidae Adams & Adams 1854
Subfamily Melanopsinae Adams & Adams 1854

#### Genus Melanopsis

Férussac, 1807

##### Type species.

*Melania costata* Olivier, 1804. Recent, Europe. Subsequent designation by Gray (1847, p. 153).

#### Melanopsis haueri ripanjensis nom. n.

Melanopsis austriaca serbica Brusina, 1902: pl. 6, figs 73–74 [non *Melanopsis serbica* Brusina, 1893].
Melanopsis haueri serbica Brusina; Wenz 1929a: 2743 [non Brusina 1893].

##### Etymology.

Named after the type locality.

##### Type locality.

Ripanj, Serbia.

##### Age.

Late Miocene (Early-Middle Pannonian; Pavlović 1927).

##### Syntypes.

Croatian Natural History Museum, Zagreb, coll. no. 2530-176/1-2 (Milan et al. 1974, p. 86).

##### Discussion.

Obviously unaware of the fact that also subspecific or variety names can constitute homonyms, Brusina (1902) introduced *Melanopsis austriaca serbica* from the Early Pannonian of Serbia, although this name was already preoccupied by another species described by himself, *Melanopsis serbica* Brusina, 1893 (p. 50). The latter species was also described from the Early Pannonian of Serbia (locality Begaljica, c. 15 km E Ripanj), but clearly represents a different taxon as evident from Brusina's descriptions and illustrations. Here we follow the taxonomic decision of Wenz (1929a), who synonymized *Melanopsis austriaca* Handmann, 1882 with *Melanopsis haueri* Handmann, 1882 (both from the Kottingbrunn, Austria) and consequently ranked the here discussed taxon as subspecies of *Melanopsis haueri*. *Melanopsis haueri serbica* can be distinguished from *Melanopsis haueri haueri* in its distinctly stronger spruce-like outline.

#### Melanopsis wolfgangfischeri nom. n.

Melanopsis rugosa [*Mel. Martiniana*] Var. *rugosa* Handmann, 1887: 26, pl. 5, figs 5–7 [non Matheron, 1842].
Melanopsis rugosa Handmann; Papp 1953: 136, pl. 10, figs 13–16 [non Matheron 1842].
Melanopsis rugosa Handmann 1887; Fischer 1996: 23 (cum syn.) [non Matheron 1842].

##### Etymology.

In honor of Wolfgang Fischer (Vienna), who greatly contributed to nomenclature and taxonomy of fossil and Recent melanopsids.

##### Type locality.

Wittmannsdorf near Leobersdorf, Austria (Fischer 1996).

##### Age.

Late Miocene (Early Pannonian, Slavonian; Papp 1951).

##### Type material.

Geological Survey Austria, Vienna, no number indicated (Fischer 1996).

##### Discussion.

This taxon is a primary homonym of *Melanopsis rugosa* Matheron, 1842 (p. 293, pl. 37, fig. 11), a fossil species from SE France. *Melanopsis rugosa* Handmann, 1887 is a member of the complexly evolving *Melanopsis impressa*-species lineage in the Late Miocene Lake Pannon (Geary 1990, Geary et al. 2012, Neubauer et al. 2013a). The morphological variability in this group resulted in the description of many names, most of which are today synonymized. While Wenz (1929a, p. 2719) regarded *rugosa* Handmann as synonym of *Melanopsis fossilis* (which is the accepted name of "*Melanopsis martiniana*"), Papp (1953), Lueger (1980) and Fischer (1996) treated it as separate species. As implied by Neubauer et al. (2013a) the validity in a biological sense of this and other species-group taxa is doubtful. Nevertheless, since many authors clearly referred to it as a separable unit, a replacement name is inevitable.

Additionally, there exists another primary homonym of *Melanopsis rugosa*, i.e. *Melanopsis lanzaeana rugosa* Brusina, 1897 from the Middle Miocene deposits of the Sinj Basin. It was synonymized with *Melanopsis lanzaeana* by Neubauer et al. (2011, p. 205), who treated it as a mere morphotype and already mentioned the problem of homonymy. We therefore avoid introducing another name for this Croatian taxon, which is not used anymore.

### Order Littorinimorpha Golikov & Starobogatov 1975
Superfamily Truncatelloidea Gray, 1840
Family Hydrobiidae Stimpson, 1865

#### Subfamily Micromelaniinae

Dybowski & Grochmalicki 1914

##### Note.

The taxonomic status of the Micromelaniinae is currently under discussion. The rank as subfamily follows Wenz (1926, p. 2126; erroneously written as "Micromelaninae"). See also Kabat and Hershler (1993) and Wilke et al. (2007). The classification of the Hydrobiidae within the Truncatelloidea follows the latest molecular systematics established by Criscione and Ponder (2013).

#### Genus Micromelania

Brusina, 1874

##### Type species.

*Micromelania cerithiopsis* Brusina, 1874. Late Miocene, Croatia. Subsequent designation by Brusina (1892, p. 164).

#### Micromelania ramacanensis nom. n.

Micromelania sp. Brusina: pl. 7, figs 59–60.
Micromelania Brusinai Pavlović, 1927: 96 [non *Micromelania brusinai* Andrusov, 1905].

##### Etymology.

Named after the type locality.

##### Type locality.

Ripanj, Ramača hamlet (also read Ramaća), Serbia.

##### Age.

Late Miocene (Early-Middle Pannonian; Pavlović 1927).

##### Syntypes.

Brusina (1902, pl. 7, figs 59–60); Croatian Natural History Museum, Zagreb, no number indicated (Milan et al. 1974).

##### Discussion.

This is a classic case of a primary homonym requiring a replacement name according to ICZN (1999, Article 57.2). *Micromelania brusinai* Andrusov, 1905 from the Maeotian of the Crimean Peninsula, Ukraine, is currently considered synonymous with *Micromelania gorianovici* Andrusov, 1897 (Davitashvili 1931, p. 27). The latter name was introduced as nomen novum by Andrusov (1897, p. 431) for the primary homonym *Micromelania striata* Andrusov, 1890 non Gorjanović-Kramberger 1890.

### Subfamily Pseudamnicolinae Radoman, 1977

#### Genus Pseudamnicola

Paulucci, 1878

##### Type species.

*Paludina macrostoma* Küster, 1853. Recent, Europe. Subsequent designation by Wagner (1928, p. 276; see also Kabat and Hershler 1993, p. 45).

#### Pseudamnicola welterschultesi nom. n.

Valvata minima Fuchs, 1877: 14, pl. 1, figs 25–27 [non *Valvata minima* Hislop, 1859].
Valvata (Cincinna) minima Fuchs; Wenz 1928a: 2439 (cum syn.) [non Hislop 1859].
Pseudamnicola minima (Fuchs, 1877); Willmann 1981: 212, textfig. 74 [non Hislop 1859].

##### Etymology.

In honor of Francisco W. Welter-Schultes (University of Göttingen), a great expert for the living non-marine mollusks of Europe.

##### Type locality.

Megara, Greece.

##### Age.

Pliocene (Papp and Steininger 1979).

##### Lectotype and paralectotypes.

Natural History Museum Vienna, coll no. 1878/0020/0023 (designation by Willmann 1981, p. 212).

##### Discussion.

This species is a primary homonym of *Valvata minima* Hislop, 1859 (p. 170, pl. 5, fig. 13) from the Tertiary of East India (see also Haszprunar 2014, p. 69) and needs a replacement name. Based on general shape and the lack of striae on the protoconch typical of *Valvata*, Willmann (1981) combined this species with *Pseudamnicola*, what is followed herein.

Jekelius (1944), Stevanović (1951) and Bartha (1955) and several other authors also documented this taxon from various localities of the early Late Miocene of Lake Pannon. Given the stratigraphical and biogeographical gaps, these records probably represent different species.

### Subfamily unknown

#### Genus Muellerpalia

Bandel, 2010

##### Type species.

*Planorbis bicincta* Fuchs, 1870 in Fuchs 1870b. Recent, Europe. Type by original designation (Bandel 2010, p. 103).

#### Muellerpalia haszprunari nom. n.

Valvata simplex Fuchs, 1870 in Fuchs 1870b: 535, pl. 21, figs 4–6 [non *Valvata tricarinata* var. *simplex* Gould, 1841].
Valvata (Valvata) simplex simplex Fuchs; Wenz 1928a: 2474 (cum syn.) [non Gould 1841].
Valvata simplex Fuchs; Strausz 1942: 80 [non Gould 1841].
Hauffenia simplex (Fuchs); Schlickum 1978: 247, pl. 18, fig. 3 [non Gould 1841].
Hauffenia simplex (Fuchs 1870); Harzhauser and Binder 2004: 9 [non Gould 1841].

##### Etymology.

In honor of Gerhard Haszprunar (Bavarian State Collection of Zoology Munich and Ludwig Maximilians University Munich), who summarized all existing names of living and fossil valvatids in a comprehensive nomenclator (Haszprunar 2014).

##### Type locality.

Tihany at Lake Balaton, Veszprém, Hungary.

##### Age.

Late Miocene (Late Pannonian, Transdanubian sensu Sacchi and Horváth 2002; Sztanó et al. 2013).

##### Type material.

According to the inventory books of the Natural History Museum Vienna the material should be stored there, but despite great effort it could not be located.

##### Discussion.

This species is a primary homonym of the extant taxon *Valvata tricarinata* var. *simplex* Gould, 1841 (p. 226) from Massachusetts, USA. The American taxon was elevated to species level by Fluck (1932). As the European species was combined with various genera since its first description and several subspecies have been described, a summary of its history is given below.

Already Schlickum (1978) considered *Valvata simplex* Fuchs, 1870 to belong to the Hydrobiidae and placed it in the genus *Hauffenia*, based on similarities of morphology and size. Recently, Bandel (2010) introduced the new genus *Muellerpalia* for *Valvata bicincta* Fuchs, 1870 in Fuchs (1870b), *Valvata carinata* Fuchs, 1870 in Fuchs (1870b), *Planorbis varians* Fuchs, 1870 in Fuchs (1870a), *Valvata simplex* Fuchs, 1870 in Fuchs (1870b), and two new species (see discussion in Neubauer et al. 2014 for the rather confusing systematics applied in Bandel 2010). We follow Bandel and place the species within *Muellerpalia*.

The following subspecies have been introduced or ranked within *Valvata simplex* Fuchs, 1870:

1) *Valvata bicincta* Fuchs, 1870 [erroneously "*bicinata*" on p. 536; from captions and description there is no doubt about the correct name] from Tihany: It was considered a subspecies of *Valvata simplex* by Lőrenthey (1906, p. 166), what was followed by Wenz (1928a, p. 2475) and Strausz (1942, p. 36). Bandel (2010, p. 103) treated it as separate species and combined it with the new genus *Muellerpalia*. Current status: *Muellerpalia bicincta*.

2) *Valvata carinata* Fuchs, 1870 (p. 536) from Tihany: It was considered as subspecies of *Valvata simplex* by Pană et al. (1981) and Pană (2003), but recombined with the new genus *Muellerpalia* by Bandel (2010, p. 104). It is, however, a primary homonym of *Valvata carinata* Sowerby, 1834 (replacement name is introduced below).

3) *Valvata simplex öcsensis* Soós, 1934 (p. 189) from Öcs: Schlickum (1978, p. 246) clearly separated this taxon from "*Hauffenia simplex*" and retained it in *Valvata*. Wenz and Edlauer (1942, p. 83) elevated it to species level, what was followed by Papp (1953, p. 109), and Harzhauser and Binder (2004, p. 10). In some of the mentioned publications the name was erroneously emended to "*oecsensis*"; the correct emendation following ICZN rules is "*ocsensis*", since it is not derived from a German expression (ICZN 1999, Article 32.5.2.1). Current status: *Valvata ocsensis*.

4) *Valvata octonaria* Brusina, 1902 (pl. 13) from Tihany: It was also ranked as subspecies of *Valvata simplex* by Wenz (1928a, p. 2476). Since it was not referred to by Bandel (2010), its generic affiliation is uncertain. Current status (needs revision): *Muellerpalia haszprunari octonaria*.

5) *Valvata simplex polycincta* Lőrenthey, 1906 (p. 167) from Tihany: It was synonymized with *Valvata simplex octonaria* by Wenz (1928a, p. 2476). Current status: junior synonym of *Muellerpalia haszprunari octonaria*.

6) *Valvata simplex unicincta* Lőrenthey, 1906 (p. 165) from Tihany (Fehérpart): The status of this taxon is doubtful. It was not mentioned by Wenz (1928a) or Bandel (2010). Given the similarity with *simplex* and *bicincta* stated by Lőrenthey, it might fall into the intraspecific variability of either of these species. Current status (needs revision): *Muellerpalia haszprunari unicincta*.

#### Muellerpalia pseudovalvatoides nom. n.

Valvata carinata Fuchs, 1870 in Fuchs 1870b: 535, pl. 21, figs 10–12 [non *Valvata carinata* Sowerby, 1834].
Valvata (Valvata) carinata Fuchs; Wenz 1928a: 2465 [non Sowerby 1834].
Valvata (Valvata) carinata Fuchs, 1870; Gillet and Marinescu 1971: 47, pl. 19, figs 10–12 [non Sowerby 1834].
Muellerpalia bicincta (Fuchs, 1870); Bandel 2010: 103, pl. 7, figs 82–85 [non *Planorbis bicincta* Fuchs, 1870 in Fuchs 1870b].

##### Etymology.

To denote that it is despite its similar shape not a member of the genus *Valvata*.

##### Type locality.

Tihany at Lake Balaton, Veszprém, Hungary.

##### Age.

Late Miocene (Late Pannonian, Transdanubian sensu Sacchi and Horváth 2002; Sztanó et al. 2013).

##### Type material.

According to the inventory books of the Natural History Museum Vienna the material should be stored there, but despite great effort it could not be located.

##### Discussion.

Up to now it has been overlooked by several authors, including ourselves (Neubauer et al. 2014), that this species is a primary homonym of *Valvata carinata* Sowerby, 1834 (see also Haszprunar 2014, p. 28). According to Bandel (2010, p. 104) this species should be classified within the new hydrobiid genus *Muellerpalia*, particularly because of its strongly different protoconch sculpture. This systematic concept is followed herein. For a more detailed discussion about the involved taxa and the species confusions in Bandel (2010) see Neubauer et al. (2014).

### Family Lithoglyphidae Tryon, 1866

#### Genus Lithoglyphus

Menke, 1830

##### Type species.

*Paludina naticoides* Pfeiffer, 1828. Recent, Europe. Subsequent designation by Herrmannsen (1846, p. 612).

#### Lithoglyphus gozhiki nom. n.

Lithoglyphus maeoticus Gozhik in Gozhik and Datsenko 2007: 88, pl. 81, figs 1–3 [non *Lithoglyphus maeoticus* Papaianopol, 2006].

##### Etymology.

In honor of Piotr F. Gozhik (Kiev), who intensively studied the Neogene deposits of Ukraine and southern Russia.

##### Type locality.

Nizhniy Dnepr (= lower Dnieper), Ukraine.

##### Age.

Late Miocene (Early Maeotian, Oltenian).

##### Holotype.

Institute of Geological Sciences, National Academy of Sciences of Ukraine, Kiev, coll. no. 2174.

##### Discussion.

A classic case of a primary homonym. Probably as a result of prolonged publication times, Gozhik had no chance to become aware of this problem. However, the taxonomic status of *Lithoglyphus maeoticus* Papaianopol, 2006 from the Early Maeotian of the Dacian Basin is doubtful. It greatly resembles and might be a synonym of the Dacian species *Lithoglyphus acutus* Cobălcescu, 1883 (p. 145, pl. 14, fig. 10; see also Wenz 1942, p. 48, pl. 15, figs 195–198).

### Clade Heterobranchia
Informal Group Lower Heterobranchia
Superfamily Valvatoidea Gray, 1840

#### Family Valvatidae

Gray, 1840

##### Note.

The here applied suprageneric systematics of *Valvata* follows Bouchet et al. (2005).

#### Genus Valvata

Müller, 1773

##### Type species.

*Valvata cristata* Müller, 1774. Recent, Europe. Type by subsequent monotypy (Müller 1774, p. 198; for details see Welter-Schultes 2012, p. 42).

#### Valvata heidemariae willmanni nom. n.

Valvata heidemariae bicarinata Willmann, 1981: 158, textfigs 56D–F [non *Valvata bicarinata* Lea, 1841].

##### Etymology.

In honor of Rainer Willmann (University of Kiel), who intensively studied the Plio-Pleistocene deposits and freshwater mollusks of Greece.

##### Type locality.

Vokasia Valley 3 km SE of Kos City, Kos Island, Greece.

##### Age.

Early Pleistocene (Middle Irakli Formation).

##### Type material.

Geological-Paleontological Institute, University of Kiel, no number indicated; Willmann (1981, textfigs 56D–E).

##### Discussion.

The species-group name *bicarinata* in combination with *Valvata* is preoccupied by the Recent species *Valvata bicarinata* Lea, 1841 from Pennsylvania, USA. The taxonomic separation from *Valvata heidemariae* Willmann, 1981 seems plausible, given the presence of a strong median keel on the upper whorl surface that is lacking in the nominal species.

### Clade Panpulmonata Jörger et al., 2010
Superorder Basommatophora Keferstein in Bronn, 1864
Order Hygrophila Férussac, 1822
Superfamily Lymnaeoidea Rafinesque, 1815
Family Lymnaeidae Rafinesque, 1815
Subfamily Lymnaeinae Rafinesque, 1815

#### Genus Radix

Montfort, 1810

##### Type species.

*Helix Auricularia* Linnaeus, 1758. Recent, Europe. Type by original designation (for details see Welter-Schultes 2012, p. 51).

#### Radix macaleti nom. n.

Radix socialis Macaleț, 2000: 252, pl. 2, figs 2–3 [non *Limnaea socialis* von Zieten, 1832].

##### Etymology.

In honor of Rodica Macaleț (Bucharest), who studied the mollusk fauna of the Dacian Basin.

##### Type locality.

Butuci near Sângeru, Prahova, Romania.

##### Age.

Latest Miocene to earliest Pliocene (Pontian, Portaferrian-Bosphorian).

##### Holotype.

Collection of the Geological Institute of Romania, coll. no. 19.546.

##### Discussion.

This species is a secondary homonym of *Limnaea socialis* von Zieten, 1832, of which the presently accepted and widely used combination is *Radix socialis* (e.g., Wenz 1923b, Gall 1972, Kókay 2006). Macaleț (2000) omitted the "sp. nov." in the heading of the description, which he indicated for all other species newly introduced by him in this paper, but gave it in the figure captions and the text and he designated a holotype. *Radix macaleti* is one of several similar species newly introduced by Macaleț (2000). Although the Lymnaeinae of the Dacian Basin are not well represented in the older literature, several of these new taxa may actually represent synonyms of one another, given the extreme variability of this clade (see, e.g., Glöer 2002, Welter-Schultes 2012). A revision of the entire group in the Dacian Basin would be necessary to clarify this issue.

### Superfamily Planorboidea Rafinesque, 1815
Family Planorbidae Rafinesque, 1815
Subfamily Planorbinae Rafinesque, 1815

#### Genus Gyraulus

Charpentier, 1837

##### Type species.

*Planorbis albus* Müller, 1774. Recent, Europe. Subsequent designation by Dall (1870, p. 351).

#### Gyraulus okrugljakensis nom. n.

Planorbis clathratus Brusina, 1884: 171, pl. 30, fig. 29 [non *Planorbis (Helisoma) clathratus* Sandberger, 1880].
Gyraulus (Gyraulus) clathratus (Brusina); Wenz 1923c: 1545 [non Sandberger 1880].

##### Etymology.

Named after the type locality.

##### Type locality.

Okrugljak (today within the city limits of Zagreb), Croatia.

##### Age.

Late Miocene (Late Pannonian, Portaferrian; Geary et al. 2010).

##### Syntype.

Croatian Natural History Museum, Zagreb, coll. no. 2953-599/1 (Milan et al. 1974, p. 117).

##### Discussion.

This species represents a primary homonym of *Planorbis (Helisoma) clathratus* Sandberger, 1880 from the Pleistocene of West Runton, Norfolk, United Kingdom. We follow Wenz (1923c), who placed Brusina's species within *Gyraulus*. The classification of the British species within *Helisoma* by Sandberger is rather doubtful. This North American genus was artificially introduced to Europe, wherefore an occurrence in the Pleistocene of the British Isles is unlikely. The morphology as depicted in Sandberger (1880) suggests an attribution to *Planorbarius*.

#### Gyraulus rasseri nom. n.

Planorbis discoideus Pavlović, 1903: 181, pl. 5, figs 14–17 [non *Planorbis multiformis discoideus* Hilgendorf, 1867].
Gyraulus (Gyraulus) discoideus (Pavlović); Wenz 1923c: 1552 [non Hilgendorf 1867].

##### Etymology.

In honor of Michael W. Rasser (State Museum of Natural History Stuttgart), who studied the *Gyraulus* species flock of Lake Steinheim.

##### Type locality.

Orahovac (= Rahovec), Kosovo.

##### Age.

Early Pliocene (Late Dacian to Early Romanian; Popović 1969).

##### Holotype.

Natural History Museum, Belgrade, coll. no. 1176 (Milošević 1962, p. 27).

##### Discussion.

The name *Planorbis discoideus* as established by Pavlović (1903) represents a primary homonym of *Planorbis discoideus* Hilgendorf, 1867. The latter species is a member of the *Gyraulus* species flock in the Middle Miocene Lake Steinheim and is presently considered a junior synonym of *Gyraulus sulcatus* by Rasser (2013). From the rather character-poor shell it is impossible to reliably attribute Pavlović's species to *Planorbis* or *Gyraulus*. Here we follow the taxonomic decision of Wenz (1923c) to place it in *Gyraulus*.

#### Gyraulus vrapceanus nom. n.

Planorbis dubius Gorjanović-Kramberger, 1890: 156, pl. 6, fig. 6 [non *Planorbis dubius* Hartmann, 1844].
Gyraulus (Gyraulus) dubius (Gorjanović-Kramberger); Wenz 1923c: 1552 [non Hartmann 1844].

##### Etymology.

Named after the type locality.

##### Type locality.

Vrapče (also read as Vrabče; today within the city limits of Zagreb), Croatia.

##### Age.

Late Miocene (Early Pannonian, Slavonian).

##### Syntype.

Croatian Natural History Museum, Zagreb, coll. no. 5195-360/2 (Milan et al. 1974, p. 119).

##### Discussion.

The name *Planorbis dubius* was first used by Hartmann (1821, p. 254) for an extant species from Zurich region in Switzerland. The name is not available from this publication, since Hartmann did not give a description or indication (see also AnimalBase project 2005–2014). He first described and thus formally introduced it in Hartmann (1844, p. 111). Today its status is disputed. Glöer (2002, p. 253) ranked it as forma within *Planorbis carinatus* Müller, 1774. Later, Glöer and Pešić (2010) stated that Hartmann's material contained two different taxa, i.e. *Planorbis planorbis* and *Planorbis carinatus*, making *Planorbis dubius* a junior synonym of both. Finally, Kantor et al. (2010) listed it as accepted species in their catalogue of Russian continental mollusks. In summary, although the status of the extant species is doubtful, the name is available. This makes *Planorbis dubius* Gorjanović-Kramberger, 1890 a primary homonym of *Planorbis dubius* Hartmann, 1844. Here follow Wenz (1923c) and classify the replacement name within *Gyraulus*.

#### Genus Planorbarius

Duméril, 1806

##### Type species.

*Helix cornea* Linnaeus, 1758. Recent, Europe. Subsequent monotypy by Froriep (1806).

#### Planorbarius halavatsi nom. n.

Planorbis grandis Halaváts, 1903: 57, pl. 3, fig. 5 [non *Planorbis grandis* Dunker in Küster et al., 1850].
Coretus grandis (Halaváts); Wenz 1923c: 1472 [non Dunker in Küster et al. 1850].
Planorbarius grandis (Halaváts); Sauerzopf 1953: 50, pl. 1, figs 3–4 [non Dunker in Küster et al. 1850].

##### Etymology.

In honor of Gyula von Halaváts (Budapest), who greatly contributed to our knowledge of Pannonian mollusks.

##### Type locality.

Balatonfőkajár, Veszprém, Hungary.

##### Age.

Late Miocene (Late Pannonian, ?Transdanubian sensu Sacchi and Horváth 2002).

##### Holotype.

Hungarian Geological Institute, Budapest, coll. no. Pl. 121 (Boda 1964, p. 130).

##### Discussion.

As both taxa were introduced within *Planorbis*, the species described by Halaváts is a primary homonym. Both are today unambiguously assigned to the genus *Planorbarius* (for the Pannonian species see, e.g., Sauerzopf 1953, Harzhauser and Tempfer 2004) and are in common usage, making the introduction of a replacement name indispensable. *Planorbis grandis* Dunker in Küster et al., 1850, an extant taxon from SE Europe, is currently ranked as subspecies of *Planorbis corneus* (see Fauna Europaea project, De Jong 2013).

#### Genus Segmentina

Fleming, 1818

##### Type species.

*Nautilus lacustris* Lightfoot, 1786 [currently considered as a synonym of *Segmentina nitida* (Müller, 1774)]. Recent, Europe. Type by monotypy (Welter-Schultes 2012, p. 70).

#### Segmentina mosbachensis nom. n.

Planorbis (Segmentina) micromphalus Sandberger, 1875: 777, pl. 33, figs 19–19c [non *Planorbis micromphalus* Fuchs, 1870 in Fuchs 1870a].
Planorbis nitidus Müll. var. *micromphalus* Sandb.; Rzehak 1888: 308 [non Fuchs 1870a].

##### Etymology.

Named after the type locality.

##### Type locality.

Mosbach, Hessen, Germany.

##### Age.

Early Pleistocene.

##### Type material.

No storage or types indicated.

##### Discussion.

The species name established by Sandberger is a primary homonym of *Planorbis micromphalus* Fuchs, 1870, although he was apparently aware of the existence of this name (compare Sandberger 1875, p. 700). Also Lőrenthey (1902, p. 190) knew about the identical naming and discussed the differences between both taxa, but did not take appropriate steps to clarify this problem. Fuchs' species was first described from the Pannonian of Rădmănești in Romania and has been recombined with *Gyraulus* by Wenz (1923c, p. 1562; see also Harzhauser et al. 2002, p. 106).

### Discussions

In the following, we present six cases of primary and secondary homonyms that seem not to be in use anymore (e.g., are unambiguously considered junior synonyms). We were unable to find any recommendation in the Code regarding the necessity of replacement names for disused junior homonyms. Following the intent expressed in Article 23.9.5, which seems to discourage the proposal of unnecessary replacement names, we choose not to introduce new names for these cases. In addition, the statuses of two taxa apparently constituting homonyms are discussed.

### Superfamily Viviparoidea Gray, 1847
Family Viviparidae Gray, 1847
Genus *Viviparus* Montfort, 1810

#### Viviparus lomejkoi brevis

Popović, 1970 non (Tournouër, 1876)

Viviparus (Viviparus) lomejkoi brevis Popović, 1970: 318, figs 1: 7, 7a, 8 [non *Paludina (Vivipara) brusinai brevis* Tournouër, 1876].

##### Type locality.

Gjurakovc (= Đurakovac), Kosovo.

##### Age.

Late Pliocene to Early Pleistocene (= "Levantin").

##### Holotype.

Collection de l'Institut de recherches géologiques et géophysiques de Belgrade, no number indicated.

##### Discussion.

Tournouër (1876) introduced *Paludina (Vivipara) Brusinai* var. *brevis* from the Early Pleistocene of Kos Island. Both genus-group names stated by Tournouër (1876) are, however, invalid. *Paludina* Férussac, 1812 is a junior objective synonym of *Viviparus* Montfort, 1810 (ICZN 1959, Op. 573) and *Vivipara* represents an incorrect subsequent spelling (Melville and Smith 1987, p. 185). The species-group name *brevis* in combination with *Viviparus* as introduced by Popović (1970) is therefore a homonym of *Viviparus brevis* (Tournouër, 1876). The latter taxon was elevated to species level by Willmann (1977); for thorough description, synonymy list, and discussion see Willmann (1981, p. 151).

Still we refrain from introducing a replacement name, because the taxonomic status of this subspecies is highly doubtful. It greatly resembles the nominal species *Viviparus lomejkoi* Pavlović, 1932 from Crmljan and Orahovac (like the type locality Gjurakovc in the Metohia Basin). The only difference is the stronger degree of whorl stepping, which is not documented by Pavlović's original description and illustrations. This is regarded to fall into intraspecific variability, why we suggest synonymizing *Viviparus lomejkoi brevis* with *Viviparus lomejkoi*. If, however, another author keeps both forms separate, a replacement name has to be introduced.

#### Viviparus berbestiensis grandis

Lubenescu & Zazuleac, 1985 non Neumayr in Herbich and Neumayr 1875

Viviparus berbestiensis grandis Lubenescu & Zazuleac 1985: 109, pl. 28, figs 15–17, pl. 29, fig. 12 [non *Vivipara grandis* Neumayr in Herbich and Neumayr 1875].
Viviparus cucestiensis grandis Lubenescu; Papaianopol and Marinescu 1995, pl. 44, fig. 5 [non Neumayr in Herbich and Neumayr 1875].

##### Type locality.

Puilor Valley, Buzău, Romania.

##### Age.

Early Pliocene (Late Dacian, Parscovian).

##### Holotype.

Institut de Géologie et Géophysique, Bucharest, coll. no. 17055.

##### Discussion.

*Vivipara*, as given by Neumayr in Herbich and Neumayr (1875, p. 413) and many other authors of this time, is an incorrect subsequent spelling of *Viviparus* Montfort, 1810 (Melville and Smith 1987, p. 185). The species-group name *grandis* in combination with *Viviparus*, as introduced for a new species by Lubenescu and Zazuleac (1985), therefore is a primary homonym of *Viviparus grandis* (Neumayr in Herbich and Neumayr 1875) and would require a replacement name (see also Wenz 1928a, p. 2323). We refrain from introducing a nomen novum because of the highly doubtful taxonomic status of this subspecies. The only criterion for Lubenescu and Zazuleac (1985, p. 110) to separate this form from the nominal species was the additional whorl and thus bigger size (therefore the name *grandis*). Apart from that it completely corresponds to *Viviparus berbestiensis* Lubenescu and Zazuleac, 1985. Consequently, we regard *Viviparus berbestiensis grandis* as junior synonym of *Viviparus berbestiensis*.

Papaianopol and Marinescu (1995) ranked *Viviparus berbestiensis grandis* as subspecies of *Viviparus cucestiensis* Lubenescu & Zazuleac 1985, but without explanation and only in the figure captions. Here we follow the original authors to avoid additional confusion.

### Superfamily Cerithioidea Fleming, 1822
Family Melanopsidae Adams & Adams 1854
Genus *Melanopsis* Férussac, 1807

#### Melanopsis pygmaea inflata

Sauerzopf, 1952 non Handmann, 1882

Melanopsis pygmaea inflata Sauerzopf, 1952: 13, pl. 2, fig. 4 [non *Melanopsis pygmaea inflata* Handmann, 1882].

##### Type locality.

No locus typicus given; occurs in Stegersbach, Litzelsdorf, Olbendorf, and Oberdorf in the Styrian Basin, Austria.

##### Age.

Late Miocene (Pannonian, Serbian, biozones E–F).

##### Type material.

No storage or types indicated (material derived from Sauerzopf's private collections).

##### Discussion.

There are several issues with the name *Melanopsis inflata*. First, the name introduced by Sauerzopf definitely constitutes a primary homonym of *Melanopsis pygmaea inflata* Handmann, 1882. Sauerzopf (1952) explicitly introduced it as new taxon, although the combination is identical to that established by Handmann. Both taxa were obviously erected for different morphologies: while Sauerzopf's form is elongated conical, Handmann's subspecies is rather globular. Handmann's taxon is meanwhile considered as junior synonym of *Melanopsis pygmaea* Hörnes, 1856 (Wenz 1929a, p. 2813). *Melanopsis pygmaea inflata* Sauerzopf, 1952, in turn, highly resembles *Melanopsis fuchsi* Handmann, 1882 concerning its size, the regular conical outline and the slightly inflated last whorl. Exactly these last two criteria were for both authors the reason to separate their forms from *Melanopsis pygmaea* (see Handmann 1887, p. 13; Sauerzopf 1952, p. 13). Therefore we consider both synonymous and refrain from introducing a replacement name.

The second problem regards the availability of *Melanopsis inflata* Handmann, 1882. This name was already introduced as subordinate taxon by Férussac (1823) within *Melanopsis buccinoidea*. Whether it is available as species-group name, however, cannot easily be determined, given the chaotic system in Férussac's work (see also discussion of *Melanopsis elongata* below) and the fact that it is not found to be used as species-group name attributed to Férussac in the literature, which would have made it available via ICZN 1999, Article 45.6.4.1. If Férussac's name is accepted as species-group name, Handmann's taxon would become a primary homonym. Since this is apparently not the case and Handmann's subspecies was synonymized anyway, the introduction of a replacement name would be inexpedient.

### *Melanopsis elongata* auctores

In the biological and palaeontological literature several species-group taxa were introduced as "*Melanopsis elongata*". The first mention traces back to Férussac (1823, p. 150), who described a subordinate taxon within *Melanopsis buccinoidea*, which he described two pages above, from Épernay, France. From Férussac's remarks it is not clear, if *elongata* has subspecific or infrasubspecific rank. Moreover, the inconsistent formatting in this work leaves doubts about what is intended to be a taxon's name and what a descriptive term. Usually it is important to find out the exact rank of a taxon, since infrasubspecific taxa are not governed by the Code. In this case, however, we follow ICZN (1999, Article 45.6.4.1), stating that an infrasubspecific taxon is deemed to be subspecific from its original publication if, before 1985, it was adopted as the valid name of a species or subspecies. This criterion is at least fulfilled by the publication of Pallary (1916).

Consequently, all later introduced taxa also named "*Melanopsis elongata*" are primary homonyms of *Melanopsis elongata* Férussac, 1923. According issues are provided by Gassies (1874, p. 384), Locard (1878, p. 58; 1893, p. 178), Doncieux (1908, p. 202), Jooss (1911, p. 72), and Gillet and Marinescu (1971, p. 55). Pallary (1916) was aware of the homonyms produced by Gassies and Doncieux and introduced the replacement names *Melanopsis goulvaini* and *Melanopsis sublongata* (erroneously written "*sublonga*" in Pallary 1926 and "*subelongata*" in Wenz 1929a). *Melanopsis elongata* Gassies, 1874 (= *Melanopsis goulvaini*) has meanwhile been synonymized with *Melanopsis frustulum* Morelet, 1857 (Bouchet 2013). Probably the problems we are presently aware of are only several of many invalidly erected taxa named "*Melanopsis elongata*".

The names introduced by Locard, Jooss and Gillet and Marinescu have not yet undergone nomenclatural revision. Although primary homonyms are invalid, it is, however, not expedient to introduce new names for taxa that are not used anymore. This particularly regards *Melanopsis narzolina elongata* Locard, 1878 from the Late Miocene of Tersanne, which was apparently not used at all by subsequent authors and synonymized by Wenz (1929a) with *Melanopsis narzolina narzolina*. If later authors regard this taxon as distinct from *Melanopsis narzolina*, a new name will have to be introduced.

A more complicated case in terms of synonymy is presented by *Melanopsis callosa elongata* Jooss, 1911 from the Aquitanian of the Mainz Basin. Wenz (1929a, p. 2729) cited the record of *Melanopsis callosa* from Jooss (1911) in the synonymy list for *Melanopsis fritzei* Thomä, 1845, both of which he considered synonymous, but either overlooked that Jooss had introduced a new variety or forgot to state it in the catalogus. The synonymization by Wenz is preliminarily accepted here, so as not to introduce yet another, probably superfluous name. A more thorough taxonomic revision is needed to clarify the taxonomic status of this subspecies and whether a new name is needed.

The remaining two homonyms are still in usage and thus require a more detailed assessment.

#### Melanopsis citharella elongata

Locard, 1893 non Férussac, 1823

Melanopsis citharella var. *elongata* Locard, 1893: 178, pl. 9, fig. 17 [non *Melanopsis elongata* Férussac, 1823].
Melanopsis citharella elongata Locard; Wenz 1929a: 2693 [non Férussac 1823].

##### Type locality.

Ueken, Aargau, Switzerland.

##### Age.

Middle to Late Burdigalian ("Helvetian").

##### Type material.

Paleontological Institute and Museum, University of Zurich, no number indicated.

##### Discussion.

Unlike the case of *Melanopsis narzolina elongata* Locard, 1878, this taxon was not synonymized by Wenz (1929a, p. 2693). Despite separating it from *Melanopsis citharella*, Wenz noted that this form is probably indistinguishable from the nominal species. After review of Locard's description and illustrations we fully agree with Wenz, and draw the taxonomic conclusion to synonymize *Melanopsis citharella elongata* with *Melanopsis citharella*. Hence, although it is a primary homonym, we avoid introducing another superfluous name.

#### Melanopsis defensa elongata

Gillet & Marinescu, 1971 non Férussac, 1823

Melanopsis defensa elongata Gillet and Marinescu, 1971: 55, pl. 23, figs 38–48 [non *Melanopsis elongata* Férussac, 1823].

##### Type locality.

Rădmănești, Romania.

##### Age.

Late Miocene (Late Pannonian, Transdanubian sensu Sacchi and Horváth 2002; Geary et al. 2010).

##### Holotype.

Gillet and Marinescu (1971) designated the specimen illustrated by Fuchs (1870a, pl. 14, fig. 79) as holotype. According to the inventory books of the Natural History Museum Vienna the material should be stored there, but despite great effort it could not be located.

##### Discussion.

This case represents another primary homonym of *Melanopsis elongata* Férussac, 1823. Here some specific notes are necessary to elucidate the history of this taxon. Gillet and Marinescu (1971) erroneously linked the holotype of *Melanopsis defensa* to the variety *trochiformis* Fuchs, 1870 (Fuchs 1870a, pl. 14, figs 77–78), who explicitly separated these two specimens from the typical form (Fuchs 1870a, p. 354). Since Fuchs did not denote a holotype, all material studied by him, except the two specimens determined as *trochiformis*, are syntypes of *Melanopsis defensa defensa*. It was unwise, though nomenclaturally correct as the nominal subspecies was still based on several (not illustrated) syntypes, to assign the new name *elongata* to the remaining figure of *Melanopsis defensa* in Fuchs (1870a, pl. 14, fig. 79). If, however, a lectotype would be designated from Fuchs's original material and one would choose the figured specimen (pl. 14, fig. 79) as such, *Melanopsis defensa elongata* would become an objective synonym of *Melanopsis defensa defensa*. In conclusion, we avoid introducing a replacement name because of the obvious misapprehension of Gillet and Marinescu (1971) and synonymize *Melanopsis defensa elongata* with *Melanopsis defensa defensa*.

A part of the material of *Melanopsis defensa defensa* in Gillet and Marinescu (1971, pl. 23, fig. 10) was later separated as the new species *Melanopsis lebedai* by Lueger (1980, p. 104).

### Order Littorinimorpha Golikov and Starobogatov, 1975
Superfamily Truncatelloidea Gray, 1840
Family Bithyniidae Gray, 1857

#### Genus Bithynia

Leach in Abel, 1818

##### Type species.

*Helix tentaculata* Linnaeus, 1758. Recent, Europe. Subsequent designation by Herrmannsen (1846, p. 114).

#### Bithynia socialis

(Papaianopol and Macaleț, 2006) non Westerlund, 1886

Bulimus (Tylopoma) socialis Papaianopol and Macaleț, 2006: 82, pl. 4, figs 1–5 [non *Bythinia socialis* Westerlund, 1886].

##### Type locality.

Bengeşti, Gorj, Romania.

##### Age.

Early Pliocene (Early Dacian, Getian).

##### Holotype.

Collection of the Geological Institute of Romania, coll. no. 18.906.

##### Discussion.

The genus-group name *Bulimus* Scopoli, 1777 was suppressed under Plenary Powers and placed on the Official Index of Rejected and Invalid Generic Names in Zoology by ICZN (1957, Op. 475). Bithyniid species originally attributed to this genus are now referred to *Bithynia* Leach, 1818. In a strict sense, this makes this species a primary homonym of *Bithynia socialis* Westerlund, 1886. Latter taxon has been recombined with *Paraelona* Beriozkina and Starobogatov in Anistratenko and Stadnichenko 1995, which is considered a junior synonym with *Bithynia* (Glöer and Maassen 2009; see also Kantor et al. 2010).

The status of *Bithynia socialis* (Papaianopol & Macaleț 2006), however, is doubtful. The bithyniids of the Dacian Basin are quite well studied (e.g., Cobălcescu 1883, Stefanescu 1896, Krejci-Graf and Wenz 1932, Wenz 1942, Pană et al. 1981), including material from the localities mentioned by Papaianopol and Macaleț (2006). The species closely resembles the co-occurring *Tylopoma speciosa* (Cobălcescu, 1883) and differs only in the stronger, rib-like growth lines, which were to a minor extent also detected for *Tylopoma speciosa* (Wenz 1942, p. 53). Therefore, we regard *Bithynia socialis* (Papaianopol & Macaleț 2006) as junior synonym of *Tylopoma speciosa* and do not introduce a nomen novum.

### Family Hydrobiidae Stimpson, 1865
Subfamily Hydrobiinae Stimpson, 1865

#### Genus Hydrobia

Hartmann, 1821

##### Type species.

*Cyclostoma acutum* Draparnaud, 1805. Recent, France. Type by monotypy.

#### Hydrobia obtusa tenuis

Wenz, 1913 non Penecke, 1886

Hydrobia obtusa [ ] mut. *tenuis* n. mut. Wenz, 1913: 113, pl. 1, figs 12–15.
Hydrobia obtusa tenuis Wenz; Wenz 1926: 1922.

##### Locality.

No type locality indicated; occurs in several places in Frankfurt am Main, Germany.

##### Age.

Early Miocene (Aquitanian, upper *Corbicula* beds = Rüssingen Formation).

##### Syntypes.

Only one of the syntypes is stored in the Senckenberg Research Institute and Natural History Museum, coll. no. SMF 245299/1 (pers. comm. R. Janssen, Frankfurt).

##### Discussion.

This tricky case requires a careful assessment of the original literature. Penecke (1886, p. 35) introduced a new species, *Hydrobia tenuis*, from the Paludina beds of Malino and Sibinj in Croatia. Later, Wenz (1913) described a different new taxon as *Hydrobia obtusa tenuis* from the Frankfurt area. Despite the identical naming, Wenz' taxon is no primary homonym. Since Wenz clearly introduced this taxon as "mutation" it is not available as species-group name (ICZN 1999, Articles 45.5 and 45.6), although he erroneously cited it as "variety" when referring to his own work in the Fossilium Catalogus (Wenz 1926). The latter record is in fact a nomen nudum (as is true for the mutations *aperta*, *distorta*, *incrassata*, and *umbilicata*). We are not aware of any work making *Hydrobia obtusa tenuis* available by treatment as valid species or subspecies (ICZN 1999, Article 45.6.4.1).

### Subfamily Pseudamnicolinae Radoman, 1977
Genus *Pseudamnicola* Paulucci, 1878

#### Pseudamnicola minima

(Lőrenthey, 1893) non (Fuchs, 1877)

Cyclostoma (?) *minima* Lőrenthey, 1893: 211, 306, pl. 4, fig. 1.
Hydrobia (Pannona) minima Lörent. sp., Lőrenthey 1902: 230, pl. 16, figs 9–11.
Amnicola (Amnicola) minima (Lőrenthey); Wenz 1926: 2068.
Pseudamnicola (Pseudamnicola) minima (Lőrenthey); Papp 1953: 117, pl. 7, fig. 10.

##### Type locality.

Șimleu Silvaniei (= Szilágy-Somlyó), Sălaj, Romania.

##### Age.

Late Miocene (Middle Pannonian, Serbian).

##### Type material.

No storage or types indicated; probably stored in the Hungarian Geological Institute, Budapest.

##### Discussion.

Both involved taxa were originally combined with different genera, but have been attributed to *Pseudamnicola* in the second half of the 20^th^ century. Lőrenthey's species was recombined with *Amnicola* by Wenz (1926), based on overall shell morphology. Because an attribution of a European species to this North American genus is relatively doubtful (Paulucci 1878, Wenz 1938–1944), Papp (1953) recombined this species with *Pseudamnicola*. *Valvata minima* Fuchs, 1877, described from the Pliocene of Megara (Fuchs 1877, p. 14, pl. 1, figs 25–27), was recombined with *Pseudamnicola* by Willmann (1981, p. 212). This would make *Pseudamnicola minima* (Lőrenthey, 1893) a secondary homonym of *Pseudamnicola minima* (Fuchs, 1877). However, as pointed out by Haszprunar (2014), *Valvata minima* Fuchs, 1877 is a primary homonym of *Valvata minima* Hislop, 1859 from the Tertiary of East India and is thus not available (for replacement name see above). Lőrenthey's species consequently is no secondary homonym and needs no replacement name. Anyway, the generic classification of neither species appears to be settled. Several species of the Miocene of Central and Southeastern Europe previously attributed to *Pseudamnicola* have been shown lately not to belong to this genus (Neubauer et al. 2013b, c).

## Supplementary Material

XML Treatment for Theodoxus

XML Treatment for Theodoxus militaris jurisicpolsakae

XML Treatment for Viviparus

XML Treatment for Viviparus stevanovici

XML Treatment for Melanopsis

XML Treatment for Melanopsis haueri ripanjensis

XML Treatment for Melanopsis wolfgangfischeri

XML Treatment for Micromelaniinae

XML Treatment for Micromelania

XML Treatment for Micromelania ramacanensis

XML Treatment for Pseudamnicola

XML Treatment for Pseudamnicola welterschultesi

XML Treatment for Muellerpalia

XML Treatment for Muellerpalia haszprunari

XML Treatment for Muellerpalia pseudovalvatoides

XML Treatment for Lithoglyphus

XML Treatment for Lithoglyphus gozhiki

XML Treatment for Valvatidae

XML Treatment for Valvata

XML Treatment for Valvata heidemariae willmanni

XML Treatment for Radix

XML Treatment for Radix macaleti

XML Treatment for Gyraulus

XML Treatment for Gyraulus okrugljakensis

XML Treatment for Gyraulus rasseri

XML Treatment for Gyraulus vrapceanus

XML Treatment for Planorbarius

XML Treatment for Planorbarius halavatsi

XML Treatment for Segmentina

XML Treatment for Segmentina mosbachensis

XML Treatment for Viviparus lomejkoi brevis

XML Treatment for Viviparus berbestiensis grandis

XML Treatment for Melanopsis pygmaea inflata

XML Treatment for Melanopsis citharella elongata

XML Treatment for Melanopsis defensa elongata

XML Treatment for Bithynia

XML Treatment for Bithynia socialis

XML Treatment for Hydrobia

XML Treatment for Hydrobia obtusa tenuis

XML Treatment for Pseudamnicola minima
